# Growth independent rhamnolipid production from glucose using the non-pathogenic *Pseudomonas putida *KT2440

**DOI:** 10.1186/1475-2859-10-80

**Published:** 2011-10-17

**Authors:** Andreas Wittgens, Till Tiso, Torsten T Arndt, Pamela Wenk, Johannes Hemmerich, Carsten Müller, Rolf Wichmann, Benjamin Küpper, Michaela Zwick, Susanne Wilhelm, Rudolf Hausmann, Christoph Syldatk, Frank Rosenau, Lars M Blank

**Affiliations:** 1 Institute for Molecular Enzyme Technology, Heinrich-Heine-University Düsseldorf, Forschungszentrum Jülich, D-52426 Jülich, Germany; 2 Institute of Applied Microbiology, RWTH Aachen University, D-52074 Aachen, Germany; 3 Laboratory of Chemical Biotechnology, TU Dortmund University, D-44227 Dortmund, Germany; 4 m2p-labs GmbH, D-52499 Baesweiler, Germany; 5 Laboratory of Biochemical Engineering, Department of Biochemical and Chemical Engineering, TU Dortmund University, D-44227 Dortmund, Germany; 6 Institute of Process Engineering in Life Sciences, Section II: Technical Biology, Karlsruhe Institute of Technology, D-76131 Karlsruhe, Germany; 7 Institute of Pharmaceutical Biotechnology, Ulm University, D-89069 Ulm, Germany

**Keywords:** flux analysis, quantitative physiology, metabolic network, biodetergent, non-pathogenic Pseudomonas, biosurfactants, rhamnolipids, off-gas analysis, ^13^C labeling, BlueSens

## Abstract

**Background:**

Rhamnolipids are potent biosurfactants with high potential for industrial applications. However, rhamnolipids are currently produced with the opportunistic pathogen *Pseudomonas aeruginosa *during growth on hydrophobic substrates such as plant oils. The heterologous production of rhamnolipids entails two essential advantages: Disconnecting the rhamnolipid biosynthesis from the complex quorum sensing regulation and the opportunity of avoiding pathogenic production strains, in particular *P. aeruginosa*. In addition, separation of rhamnolipids from fatty acids is difficult and hence costly.

**Results:**

Here, the metabolic engineering of a rhamnolipid producing *Pseudomonas putida *KT2440, a strain certified as safety strain using glucose as carbon source to avoid cumbersome product purification, is reported. Notably, *P. putida *KT2440 features almost no changes in growth rate and lag-phase in the presence of high concentrations of rhamnolipids (> 90 g/L) in contrast to the industrially important bacteria *Bacillus subtilis, Corynebacterium glutamicum*, and *Escherichia coli. P. putida *KT2440 expressing the *rhlAB*-genes from *P. aeruginosa *PAO1 produces mono-rhamnolipids of *P. aeruginosa *PAO1 type (mainly C_10_:C_10_). The metabolic network was optimized in silico for rhamnolipid synthesis from glucose. In addition, a first genetic optimization, the removal of polyhydroxyalkanoate formation as competing pathway, was implemented. The final strain had production rates in the range of *P. aeruginosa *PAO1 at yields of about 0.15 g/g_glucose _corresponding to 32% of the theoretical optimum. What's more, rhamnolipid production was independent from biomass formation, a trait that can be exploited for high rhamnolipid production without high biomass formation.

**Conclusions:**

A functional alternative to the pathogenic rhamnolipid producer *P. aeruginosa *was constructed and characterized. *P. putida *KT24C1 pVLT31_*rhlAB *featured the highest yield and titer reported from heterologous rhamnolipid producers with glucose as carbon source. Notably, rhamnolipid production was uncoupled from biomass formation, which allows optimal distribution of resources towards rhamnolipid synthesis. The results are discussed in the context of rational strain engineering by using the concepts of synthetic biology like chassis cells and orthogonality, thereby avoiding the complex regulatory programs of rhamnolipid production existing in the natural producer *P. aeruginosa*.

## Background

Rhamnolipids are biosurfactants with high industrial potential. The possible applications are manifold, as reviewed by Lang and Wullbrandt [1] and Maier and Soberón-Chávez [2]. For example, rhamnolipids can be used as detergents in washing agents due to their tensio-active properties. They can also be used as emulsifiers in the cosmetic and food industry [3,4]. Particularly, they present a good alternative to chemically synthesized detergents, because they are biodegradable, show novel properties like antimicrobial effects [5-7], and are produced from renewable resources.

Rhamnolipids are mainly produced by bacteria of the genus *Pseudomonas*; with most reports focusing on strains of the opportunistic pathogen *P. aeruginosa*. Rhamnolipids are composed of one or two hydrophobic β-hydroxy fatty acids, which are linked through a β-glycosidic bond to one or two rhamnose molecules [8,9] forming the hydrophilic moiety. According to the number of rhamnose moieties, mono- and di-rhamnolipids are differentiated. The fatty acids alkyl chain length in *P. aeruginosa *can vary from C_8 _to C_14 _[10]; the most abundant species contains two β-hydroxy fatty acids with C_10 _chains [8]. The alkyl chains can also contain up to two unsaturated C-C bonds [5].

The rhamnose moiety is synthesized from glucose-6-phosphat in five consecutive enzymatic steps [11]. The β-hydroxy fatty acids in turn originate from fatty acid de novo synthesis [12]. Both metabolic pathways exist in many Gram-negative bacteria; besides de novo fatty acid synthesis, the rhamnose synthesis pathway provides building blocks for lipopolysaccharides of the outer membrane of these organisms [13,14].

**Figure 1:**
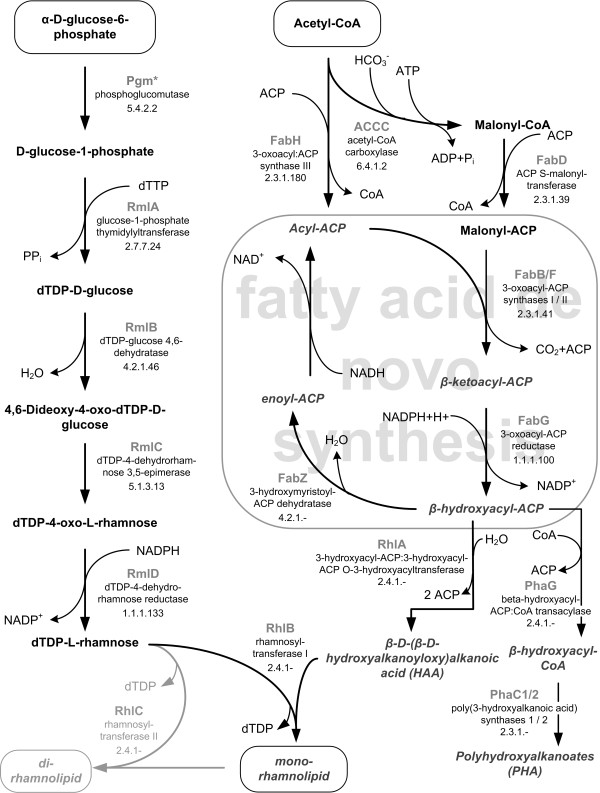
**Rhamnolipid biosynthesis pathway in *P. putida***. All enzymatic steps required for the synthesis of essential precursors including host-intrinsic enzymes and exogenous RhlA and RhlB are shown. *) Annotated in *P. putida *as XanA [31].

The synthesis pathway of rhamnolipids consists of three dedicated enzymatic reactions. In the first step two activated β-hydroxy fatty acids are linked by RhlA, the 3-hydroxyacyl-ACP (acyl carrier protein):3-hydroxyacyl-ACP O-3-hydroxyacyltransferase, to a dimer, called 3-(3-hydroxyalkanoyloxy)alkanoate (HAA) [12,15]. Mono-rhamnolipids are produced by RhlB, the rhamnosyltransferase I, by the condensation of HAA and dTDP-L-rhamnose [16,17]. The rhamnosyltransferase II (RhlC) adds another rhamnose moiety to the mono-rhamnolipid resulting in a di-rhamnolipid [17] (Figure 1). Notably, RhlG, a β-hydroxyacyl-ACP:CoA (coenzyme A) transacylase, previously associated with rhamnolipid synthesis, is not required for rhamnolipid synthesis in vitro [12,18]. The genes *rhlA *and *rhlB *are organized in a single operon, while *rhlC *is localized in another region of the *P. aeruginosa *genome and forms an operon with a gene of unknown function.

In *P. aeruginosa *the expression of all three genes involved in rhamnolipid synthesis is regulated by two quorum sensing systems and is active when *P. aeruginosa *is cultivated under phosphate or nitrogen limiting conditions [6,19-21]. The σ^54 ^factor is responsible for the expression of *rhlAB *under these conditions [22]. LasI/R and RhlI/R are also regulating transcription of many virulence factors [23].

*P. aeruginosa *has the highest rate of rhamnolipid production on plant oils as sole energy and carbon source [6,24]. The use of plant oils in industrial rhamnolipid production is a major drawback, as rhamnolipids are strong emulsifiers requiring elaborate and expensive methods for product purification, including organic solvent intensive extraction [20,25].

There are however approaches to produce rhamnolipids in bacteria other than *P. aeruginosa *by heterologous gene expression. Recombinant rhamnolipid production generally enables industrial important advantages. They facilitate both disconnecting biosynthesis from the complex quorum sensing regulation and the opportunity of avoiding pathogenic production strains, in particular *P. aeruginosa*. A topical review on rhamnolipid production by genetic engineering strategies using *P. aeruginosa rhl-*genes is given by Toribio *et al*. [26]. For example, Ochsner *et al*. [27] examined rhamnolipid synthesis by recombinant *P. fluorescens, P. putida, P. oleovorans*, and *E. coli*, which were equipped with the *rhlAB *operon. Recombinant strains of *P. fluorescens *were able to produce 0.25 g/L, those of *P. putida *0.6 g/L rhamnolipid. Active rhamnosyltransferase was also synthesized in *E. coli *and *P. oleovorans*, but no rhamnolipids were formed. Wang *et al*. [28] reported that heterologous expression of the *rhlAB *genes in the *E. coli *strains BL21 and TnERAB led to the formation of rhamnolipids. Cabrera-Valladares *et al*. [29] also examined the heterologous production of mono-rhamnolipids in *E. coli *W3110. They constructed an *E. coli *strain that expressed the *P. aeruginosa *operons *rhlAB *and *rmlBDAC*. The latter encodes the dTDP-L-rhamnose synthesis pathway. With glucose as sole carbon source, they achieved a concentration of 120 mg/L mono-rhamnolipid and were able to show that recombinant production of mono-rhamnolipid in *E. coli *is limited by the availability of dTDP-L-rhamnose. Cha *et al*. [30] examined the option of expressing *rhlAB *from *P. aeruginosa *in *P. putida*. They were able to produce up to 7.3 g/L rhamnolipid with a recombinant *P. putida *strain, utilizing soybean oil as substrate. Another study with soybean oil as carbon source was carried out by Wang *et al*. [28]. They inserted the *rhlAB *genes into *P. aeruginosa *PEER02 through transposon-mediated chromosome integration; the resulting strain produced 1.8 g/L rhamnolipid. Cabrera-Valladares *et al*. [29] also used a hydrophobic substance (oleic acid) as substrate and detected 52 mg/L rhamnolipid with recombinant *E. coli *HB101.

In the present study, we designed, constructed, and analyzed a mono-rhamnolipid producing *P. putida *KT2440 that overcomes the outlined limitations of the natural producer *P. aeruginosa*. The constructed strain is devoid of pathogenicity factors [31] and complex cellular regulation, and uses glucose as carbon source thereby avoiding extensive downstream processing. The results are discussed in the context of different approaches of heterologous rhamnolipid production.

## Results

### High rhamnolipid resistance as prerequisite for the production host

**Figure 2:**
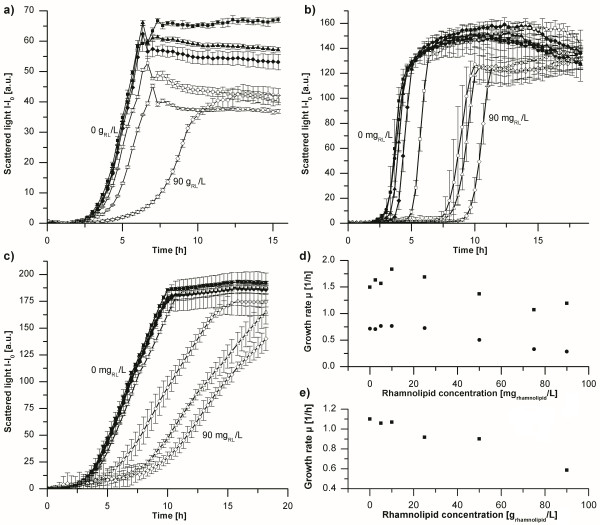
**Toxicity studies with di-rhamnolipids using different bacteria**. (a) Inhibiting effect of di-rhamnolipids on *E. coli*. Di-rhamnolipid concentrations tested were 0 g/L (■), 5 g/L (▲), 10 g/L (♦), 25 g/L (□), 50 g/L (○), and 90 g/L (◊). (b) Inhibiting effect of di-rhamnolipids on *B. subtilis*. Di-rhamnolipid concentrations tested were 0 mg/L (■), 2.5 mg/L (**●**), 5 mg/L (▲), 10 mg/L (♦), 25 mg/L (□), 50 mg/L (○), 75 mg/L (Δ), and 90 mg/L (◊). (c) Inhibiting effect of di-rhamnolipids on *C. glutamicum*. Di-rhamnolipid concentrations tested were 0 mg/L (■), 2.5 mg/L (**●**), 5 mg/L (▲), 10 mg/L (♦), 25 mg/L (□), 50 mg/L (○), 75 mg/L (Δ), and 90 mg/L (◊). (d) Growth rates resulting from toxicity experiments with *B. subtilis *(■) and *C. glutamicum *(**●**). (e) Growth rates resulting from toxicity experiments with *E. coli*.

The non-pathogenic host for rhamnolipid production from glucose has to withstand high rhamnolipid concentrations to sustain industrially relevant production titers. The industrial workhorses *E. coli, B. subtilis*, and *C. glutamicum*, as well as the closely related, but non-pathogenic *Pseudomonas, P. putida *were tested for resistance against di-rhamnolipids. Using a microbioreactor system, the growth of the species was monitored (Figure 2) in the presence of up to 90 g/L di-rhamnolipid (purity of 95%).

The effect of rhamnolipids on Gram-positive *C. glutamicum *is dramatic, where concentrations of less than 100 mg reduce the rate of growth by 60% (Figure 2d). *B. subtilis *on the other hand, being Gram-positive as well, features only slightly decreased growth rates, while the duration of the lag-phase increases significantly in the presence of rhamnolipids (Figure 2b). *B. subtilis *secretes lipases, which might be able to disassemble di-rhamnolipid, by splitting off the fatty acids from the sugar molecules, reducing the toxic effect significantly. Then, *B. subtilis *would be able to grow unaffected by the rhamnolipids derived residues, explaining the elongated lag-phase and the almost unimpaired growth rates. Possible candidates that might extracellularly digest rhamnolipids are the enzymes lipoyl synthase (EC 2.8.1.8) and triacylglycerol lipase (EC 3.1.1.3), being products of *lipA *and *lipB*, respectively [32].

In contrast, Gram-negative species appear to be less affected and can grow in the presence of highly concentrated rhamnolipids. At a concentration of 90 g/L di-rhamnolipid *E. coli *only grows with half the growth rate it features when growing in absence of rhamnolipids (Figure 2e). Importantly, *P. putida *KT2440, certified as a safety strain [31,33] showed little change in growth rate in dependence of rhamnolipid concentrations as high as 90 g/L (data not shown). As this strain is closely related to *P. aeruginosa*, contains both necessary pathways for rhamnolipid precursor synthesis (i.e., de novo lipid synthesis and creation of activated rhamnose) [31], and grows with a very high rate on glucose [34], *P. putida *KT2440 was chosen as host for rhamnolipid production using glucose as carbon source.

### Blueprint of an optimal metabolic network for rhamnolipid production

Having the chassis, the design of a metabolic network with high capacity for rhamnolipid synthesis in *P. putida*, using flux balance analysis with the rate of rhamnolipid production as linear programming objective, was in focus. The constraints of the metabolic network were, besides its structure, the substrate uptake rate, the rate of biomass formation, and the energetic demand for cell maintenance.

**Figure 3:**
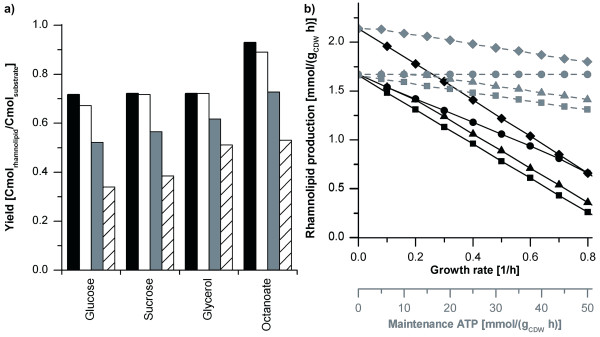
**Theoretical yields in rhamnolipid production with *P. putida *under different conditions**. (a) Rhamnolipid yields on alternative carbon substrates. The yields were calculated for zero growth (black bars), zero growth and 30 mmol ATP/(g_CDW _h) maintenance metabolism (white bars), growth at a rate of 0.4 1/h with 30 mmol ATP/(g_CDW _h) maintenance metabolism (gray bars), and a growth rate of 0.8 1/h with 50 mmol/(g_CDW _h) maintenance metabolism (shaded bars). The substrate uptake rates were constrained to 120 mCmol/(g_CDW _h). (b) Rhamnolipid production capacity depending on biomass formation and maintenance metabolism for alternative carbon substrates. The course of rhamnolipid production for uptake of glucose (■), glycerol (●), sucrose (▲) and octanoate (♦) are displayed. The black curves display rhamnolipid production in dependence on the rate of growth; grey dashed curves display rhamnolipid production in dependence of maintenance metabolism.

**Figure 4:**
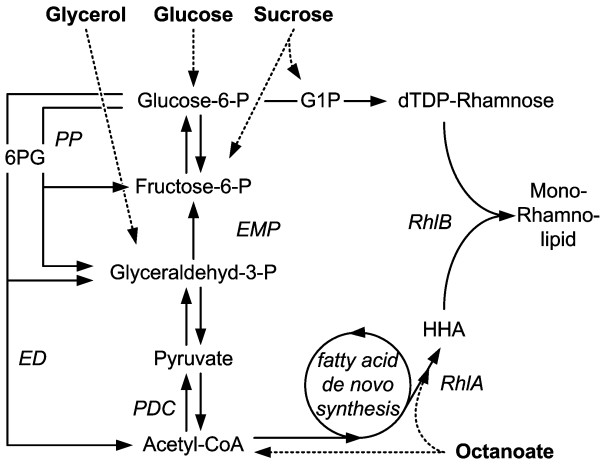
**Scheme of utilized pathways during rhamnolipid production by *P. putida***. ED, Entner-Doudoroff pathway; PP, pentose phosphate pathway; PDC, pyruvate decarboxylation; G1P, glucose-1-phosphate; HAA, β-D-(β-D-hydroxyalkanoyloxy)alkanoic acid; RhlA, 3-hydroxyacyl-ACP:3-hydroxyacyl-ACP O-3-hydroxyacyltransferase; RhlB, rhamnosyltransferase I.

The theoretically achievable yields of rhamnolipids on industrial relevant substrates (glucose, sucrose, glycerol, and fatty acids (here as example octanoate)) were estimated. The computational results indicate that cell growth should be minimized to achieve high rhamnolipid yields (Figure 3). In simulations without growth and no or low cell maintenance, rhamnolipid yields varied only slightly between the two sugars and glycerol. The choice of substrate had an effect during growth or high cell maintenance metabolism. Specifically, sucrose and glycerol simulations were superior to simulations with glucose, as the glucose ABC-transporter requires one molecule of ATP per glucose molecule transported, while glycerol is transported via an ion channel by diffusion [31,35]. *P. putida *does not feature a sucrose uptake system, which therefore has to be established by simulating a sucrose porin channel [36] present in some *P. syringae *strains [37,38]. According adaptations of the computational metabolic network yielded in a non-cellular energy consuming sucrose uptake. In subsequently carried out simulations, octanoate enabled the highest yield (Figure 3b). Assuming the presence of an ACP-ligase [39], octanoate requires only activation by CoA/ACP to form HAA (Figure 4), thereby avoiding metabolically expensive de novo lipid synthesis. The rhamnose moiety requires β-oxidation and gluconeogenic reactions.

Notably, in simulations with low growth rates and maintenance metabolism, rhamnolipid yield on glycerol equaled yields on sugars; with higher growth rate and maintenance metabolism, the yield on glycerol equaled the yield on octanoate. The observation that a more reduced carbon substrate is not beneficial for rhamnolipid production by non-growing cells suggests that under these conditions a carbon limitation exists, while production by growing cells can be energy limited. Glycerol feeds into the central carbon metabolism at the level of glyceraldehyde-3-phosphate and thus does not utilize the pentose phosphate (PP) pathway, which wastes carbon via CO_2 _production (Figure 4). Glucose and sucrose enter the central carbon metabolism via the Entner-Doudoroff (ED) pathway. To supply the cell with the necessary energy for maintenance (via redox cofactor synthesis), the PP pathway was active, hence wasting carbon via CO_2 _formation. In scenarios with very high energy demand (i.e., high growth rates and/or high maintenance metabolism), full oxidation via acetyl-CoA and the tricarboxylic acid (TCA) cycle was observed, again resulting in CO_2 _formation and concomitant lower rhamnolipid yield. The theoretically high rhamnolipid yield on octanoate is a result of the omitted reaction from pyruvate to acetyl-CoA (via the pyruvate dehydrogenase).

In summary, the carbon substrates used for rhamnolipid production by *P. aeruginosa *are theoretically ideal for achieving high yields of product. However, the extensive product purification from a second, hydrophobic phase is not desirable. Therefore, the industrially important carbon source glucose is preferred. It is desirable to produce rhamnolipids from glucose with a host that has low maintenance requirements and forms no side-products. Ideally, such a host allows production during non-growth conditions, to maximize the yield of product on substrate.

Having the carbon substrate defined, subsequent simulations focused on a metabolic network that is optimal for rhamnolipid production from glucose. Possible reaction candidates include glucose uptake and glucose catabolism. The earlier via a newly introduced phosphotransferase system to improve the stoichiometry of glucose uptake and the latter via an artificial Embden-Meyerhof-Parnas (EMP) pathway by introducing a phosphofructokinase to improve ATP generation by substrate level phosphorylation, respectively. Notably, the improvements of the tested scenarios were minor (below 1% of additional rhamnolipid) again highlighting that carbon and not energy availability determines the yield of rhamnolipid production. Hence, these optimizations were not considered as valuable targets for the improvement of rhamnolipid production. Instead, removing enzymatic reactions that are not necessary for rhamnolipid production, but potentially waste carbon moved into focus. As already mentioned, no side products from central carbon metabolism (e.g., carboxylic or amino acids) were observed in the growth medium. Therefore, the avoidance of biomass components that are not necessary for survival and production are the only means for metabolic optimization. One such target is the storage polymer polyhydroxyalkanoate (PHA). In *P. putida*, PHA-synthesis directly competes with the production of the rhamnolipid precursor HAA (Figure 1). Eliminating this reaction in vivo should lead to enhanced rhamnolipid yields. Choi *et al*. [40] recently showed in *P. aeruginosa *PA14 and PAO1 Δ*phaC1 *mutants that indeed PHA-production was lower. Again, after reaching a defined concentration of cells in the media, which suits rhamnolipid production best, biomass formation should be deactivated or avoided to enable optimal carbon exploitation.

### Rhamnolipid production from glucose by non-pathogenic *P. putida*

**Figure 5:**
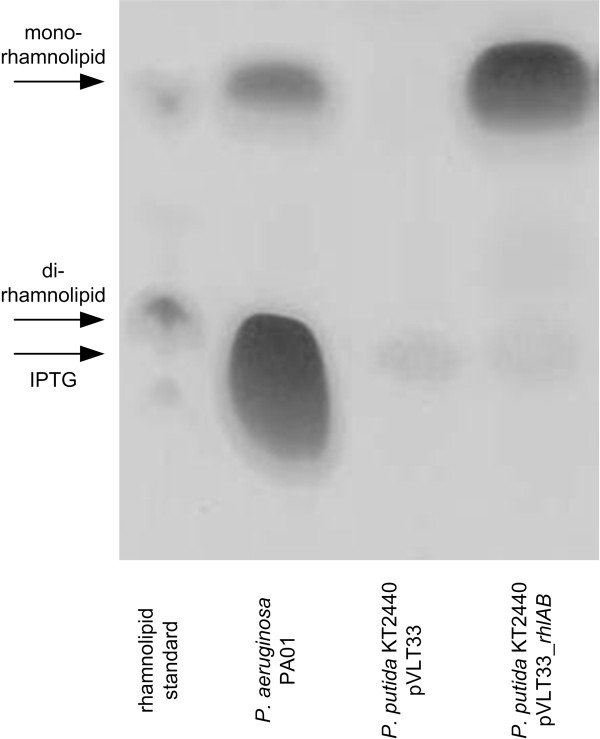
**Thin layer chromatography of rhamnolipids**. The sample of *P. aeruginosa *PAO1 (lane 2), grown in PPGAS-medium at 37°C, contains mono- and di-rhamnolipid as does the commercial rhamnolipid (JBR425, Jeneil Biosurfactant Co.) (lane 1). *P. putida*, expressing the *rhlAB *operon of *P. aeruginosa *from the plasmid pVLT33_*rhlAB*, cultivated in glucose containing LB-medium produced mono-rhamnolipid (lane 4). Empty vector control pVLT33 (lane 3). The band located above the di-rhamnolipids is IPTG (i.e., lanes 3 and 4).

To enable the production of rhamnolipids in *P. putida*, the essential genes for rhamnolipid production, located in *P. aeruginosa *on the *rhlAB *operon (encoding RhlA and RhlB) were introduced. The resulting recombinant strain *P. putida *KT2440 pVLT33_*rhlAB *produced mono-rhamnolipids (Figure 5). In comparison, *P. aeruginosa *PAO1 produced mono- and di-rhamnolipids (Figure 5, lane 2). First quantification of rhamnolipids indicated up to 0.35 g/L rhamnolipid in the *P. aeruginosa *culture, and about 0.22 g/L mono-rhamnolipid in the culture of the newly engineered *P. putida *KT2440 pVLT33_*rhlAB*.

**Figure 6:**
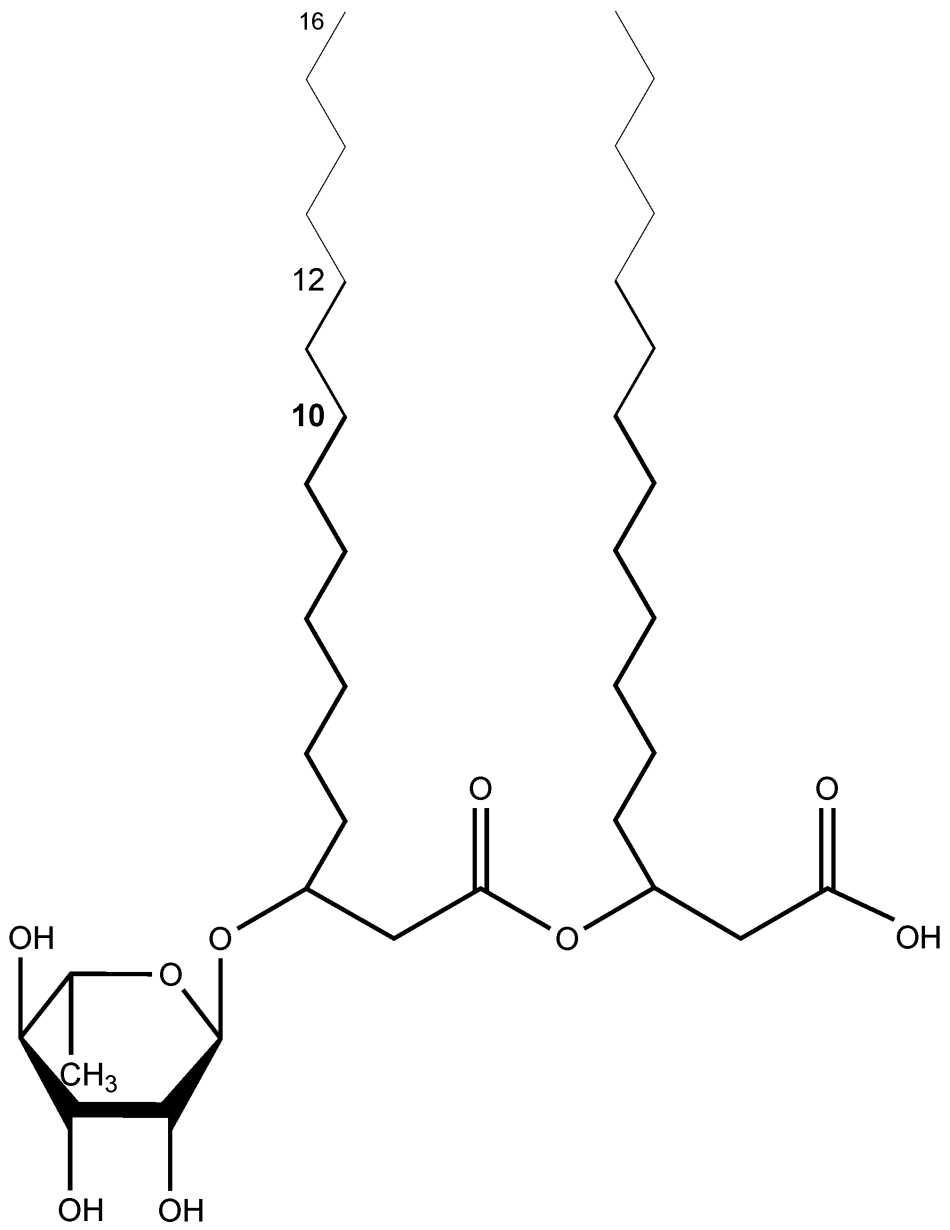
**Molecular structure of mono-rhamnolipids from recombinant *P. putida***. The major compound (C_10_:C_10_) is indicated by thick black lines, while minor compounds are indicated by thin lines.

The product spectrum of *P. putida *KT2440 pVLT33_*rhlAB *was investigated by HPLC-ESI-MS. The results illustrated that the new strain produces rhamnolipids with fatty acids featuring chain lengths between C_8 _and C_12 _in different combinations and in addition very low amounts of rhamnolipids with C_14 _and C_16 _chains. Furthermore, some of the alkyl chains contained one unsaturation. The most abundant rhamnolipid species contained two fatty acids with C_10 _chains (Figure 6). This was also the case for rhamnolipids that contained only one β-hydroxy fatty acid chain. Although *P. aeruginosa *and *P. putida *KT2440 pVLT33_*rhlAB *produced rhamnolipids with different number of rhamnose residues, the produced rhamnolipids consisted of alkyl chains with the same length. Hence, the production of rhamnolipid with similar composition to the rhamnolipid of *P. aeruginosa *(*rhlC *addition leads to di-rhamnolipid synthesis) using glucose as carbon source was achieved.

### Rhamnolipid synthesis by a PHA-negative mutant

**Figure 7:**
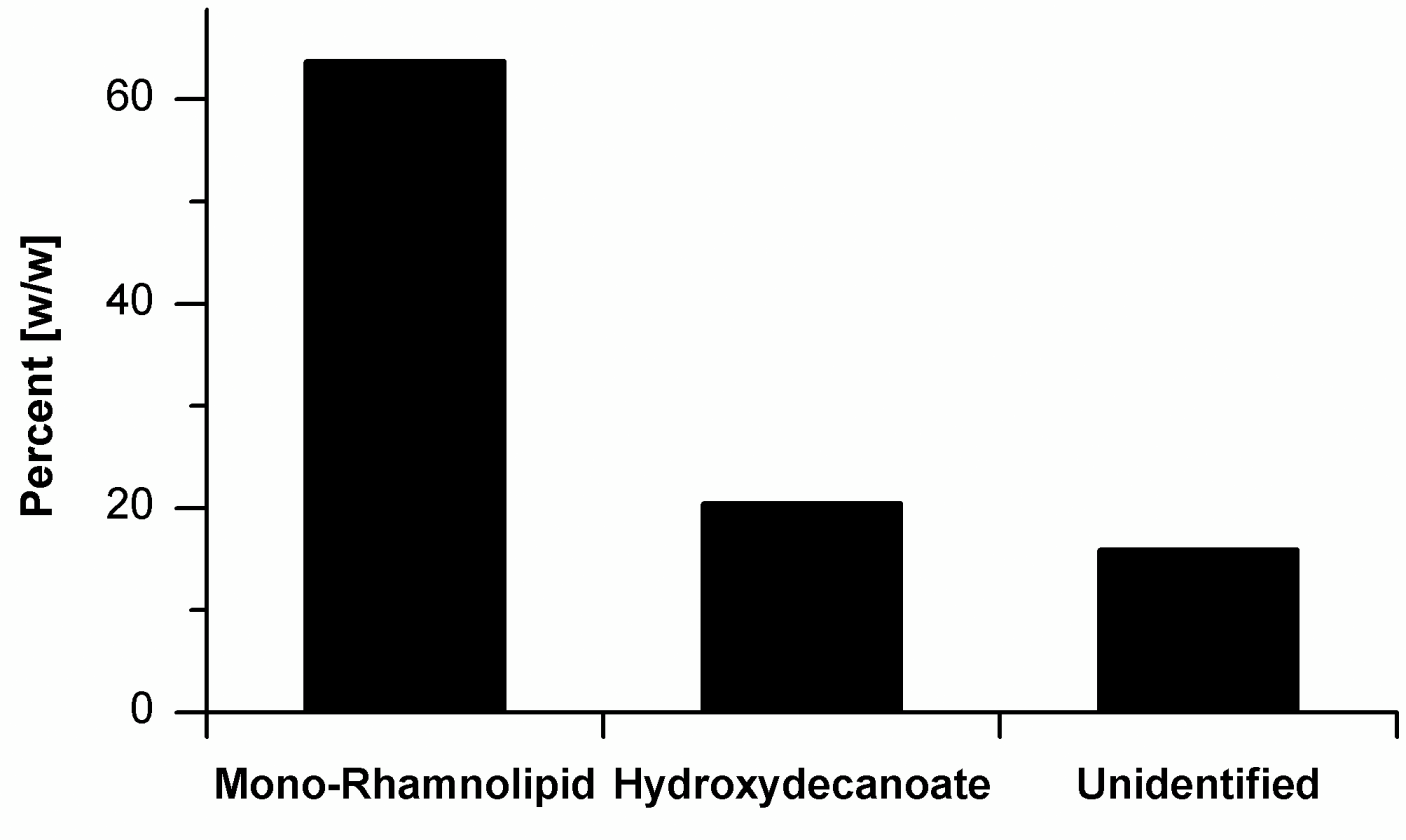
**Percentage distribution of compounds gained by hydrophobic adsorption from the supernatant of a fermentation of *P. putida *KT42C1 pVLT31_*rhlAB***.

While it was shown that the engineered *P. putida *KT2440 pVLT33_*rhlAB *produced rhamnolipids, the yield on glucose was significantly below the theoretical limit of 0.72 Cmol_rhamnolipid_/Cmol_glucose _(Figure 3). The identified PHA production pathway was chosen as potentially valuable target. *P. putida *uses β-hydroxyacyl-ACP from de novo fatty acid synthesis as precursor for PHA storage [41-43]. The β-hydroxyacyl-ACP is converted to β-hydroxyacyl-CoA by PhaG β-hydroxyacyl-ACP:CoA transacylase [44]. PhaC1 poly(3-hydroxyalkanoic acid) synthase 1 catalyzes the reaction leading to PHA [45]. As these reactions compete with formation of hydroxyalkanoyl-alkanoates in the rhamnolipid synthesis pathway, the use of a PhaC1-negative strain was chosen as first optimization target. *P. putida *KT42C1 (Δ*phaC1*::Km^r^) [41], a derivative of *P. putida *KT2442 [46], was tested as a host for rhamnolipid production. *P. putida *KT2442 in turn is a spontaneous rifampicin resistant mutant of *P. putida *KT2440. As the knockout was produced using a kanamycin resistance cassette, the *rhlAB *operon was subcloned into the plasmid pVLT31, a pVLT33 derivative carrying a tetracycline resistance. The new strain *P. putida *KT42C1 pVLT31_*rhlAB *produced up to 1.5 g/L of rhamnolipid, about seven times more than the original strain. The product profile of *P. putida *KT42C1 pVLT31_*rhlAB *revealed that not only mono-rhamnolipids, but also up to 20% of the free hydroxy fatty acid was produced (Figure 7). To avoid wasting of resources the formation of the hydroxy fatty acids should be minimized. Therefore, future experiments concentrate on the two alternative reactions forming the free fatty acids: de novo synthesis or rhamnolipid degradation. Also conceivable would be the knocking out of the gene for the second poly(3-hydroxyalkanoic acid)synthase *phaC2*, which features minor PHA-producing activities [41].

### Uncoupling growth and rhamnolipid production: towards minimal biomass formation

**Figure 8:**
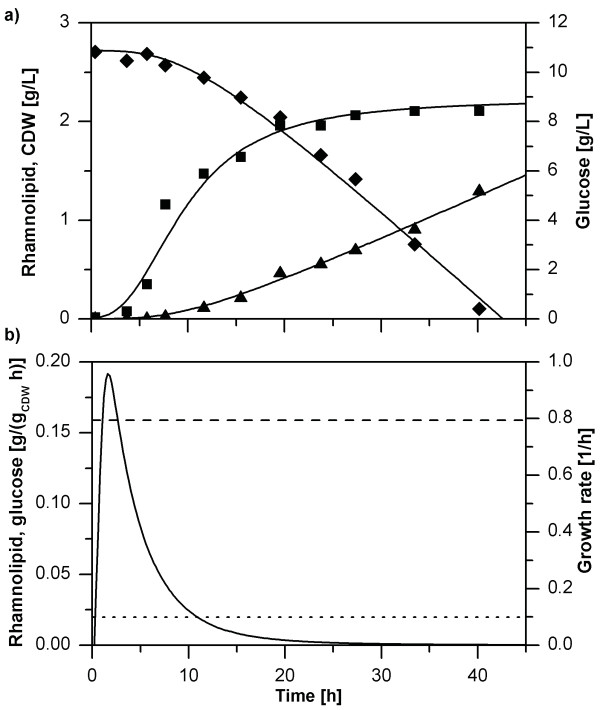
**Uncoupling of rhamnolipid production and cell growth of *P. putida *in a 50 mL baffled flask**. (a) Fermentation characteristics including cell growth (■) and course of rhamnolipid (▲) and glucose (♦) concentrations and their respective fitted courses. CDW, cell dry weight. (b) Specific rates resulting from the fitted experimental data. The black line represents the course of the growth rate, while the dashed line and the dotted line show the specific glucose uptake rate and the specific rhamnolipid production rate respectively.

Ideally, to achieve optimal product yields, high production rates without biomass formation are desirable. During the fermentation, the growth rate of *P. putida *KT42C1 pVLT31_*rhlAB *declined (Figure 8). This growth behavior on lysogeny broth (LB) medium was described for *E. coli *and is explained by multiauxic growth due to sequential compound uptake [47,48]. The kinetics underlying growth of *P. putida *can best be described by a logistic growth formulation (Equations (1) to (3)).

Although counterintuitive, changing growth rates did not result in changing neither glucose uptake nor rhamnolipid production rates (Figure 8). Importantly, the constant rhamnolipid formation rate suggests that the recombinant regulation of the rhamnolipid synthesis operon cannot be influenced by the host and hence is truly orthogonal during the growth and production phases. Hence, rhamnolipids are produced from cells that grow minimally or not at all, which opens opportunities for long-term stable production with high product yields (Figure 3). This is especially true, if glucose is the carbon source for rhamnolipid production, while the components of the LB medium are precursors for biomass formation.

To elucidate if glucose primarily serves as substrate for rhamnolipid production by *P. putida *KT42C1 pVLT31_*rhlAB *the use of glucose versus the use of alternative carbon sources was discriminated using uniformly labeled ^13^C_6_-glucose in combination with appropriate analytics. A new custom-made off-gas sensor (BlueSens GmbH, Herten, Germany) allowed the simultaneous quantification of ^12^CO_2 _and ^13^CO_2 _concentrations. Assuming that glucose is the carbon source for rhamnolipid production, the conversion of pyruvate to acetyl-CoA (the monomer of fatty acid polymerization) releases ^13^CO_2_.

**Figure 9:**
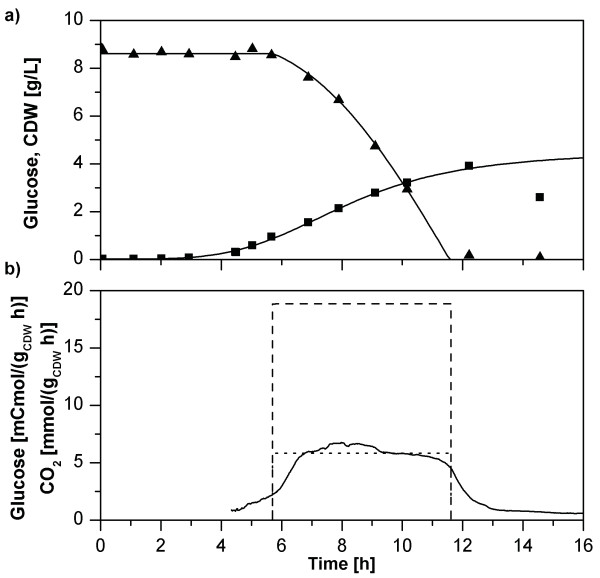
**Kinetics of rhamnolipid-production in *P. putida *in a stirred 300 mL reactor**. (a) Development of biomass (■) and glucose (▲) concentrations. The experimental data is depicted by symbols, while the lines present the fits using Equations (1) to (3). CDW, cell dry weight. (b) Specific rates characterizing rhamnolipid production in *P. putida*. The solid line presents the measured ^13^CO_2 _production rate originating from ^13^C-labeled glucose. The dashed line represents the glucose uptake rate and was calculated from the fitted glucose concentration curve. The dotted line shows the rate of CO_2 _evolution, produced in the rhamnolipid synthesis pathway. The curve was calculated from the experimentally determined yield of rhamnolipid on glucose and the estimated rate of glucose consumption.

The production of ^13^CO_2 _did not agree with time-invariant glucose uptake and rhamnolipid production rates, but rather a distinct and abrupt start of glucose metabolization followed by a constant ^13^CO_2 _production of 0.21-0.27 mmol/(g_CDW _h) (CDW: cell dry weight). The specific production rate of ^13^CO_2 _remained constant until glucose was depleted (Figure 9). The growth and glucose uptake kinetics suggest that glucose metabolism is suppressed by LB constituents, followed by glucose catabolism after these constituents are depleted, i.e., multiauxic growth [49]. Rhamnolipid production on glucose-complemented LB medium can be divided into two phases, of which the first consists of rapid growth on the most favored carbon substrates contained in the LB medium, while the second phase is characterized by a decreasing growth rate at constant specific glucose uptake and rhamnolipid production rates, i.e., by gradual uncoupling of biomass and rhamnolipid production.

The new off-gas sensor from BlueSens allowed to rationalize these observed fermentation kinetics and provided valuable insights into metabolic network operation, which is of paramount importance for the development of rhamnolipid production by *P. putida *KT42C1 pVLT31_*rhlAB *under non-growth conditions.

## Discussion

### Comparison to *P. aeruginosa *wild type strains

Until now, rhamnolipid production mainly was carried out by *P. aeruginosa*. In this work, a functional substitution to the opportunistic pathogenic, nosocomial *P. aeruginosa*, a non-pathogenic *P. putida *was developed. The engineered strain has several advantages when compared to rhamnolipid production with *P. aeruginosa*. We were thus for instance able, to circumvent the complex quorum sensing regulation in *P. aeruginosa*.

The achieved rhamnolipid production rate in the carried out experiments amounted to two thirds of the rate observed in optimized fermentations with *P. aeruginosa *(Table 1). The obtained carbon yield also was in the same range. For industrial production of rhamnolipids the yield is an important parameter, as it determines the expenses for the substrate. The rate of rhamnolipid production might be raised by increasing activated rhamnose availability as observed earlier [29]. Nevertheless, the obtained rhamnolipid titer in the medium is still far from the almost 40 g/L rhamnolipid reported with *P. aeruginosa *[20], which can be explained by the low biomass concentration in the described experiments. Fortunately, *P. putida *is suited for high cell density fermentations. While Kim *et al*. achieved 100 g_CDW_/L with *P. putida *BM01 [50], even higher concentrations were obtained with *P. putida *KT2442 [51]. In another study, with *P. putida *KT2440, cell concentrations of up to 62 g_CDW_/L were achieved [52].

**Table 1:** Recombinant rhamnolipid production

Organism	Medium	Substrates		Cell Dry Weight	Carbon Yield^1^	Maximal Titer	Process Time	RL-Production Rate	Space-Time Yield	Specific RL-Production Rate^2^	Reference
		Substance	[g/L]	[g_CDW_/L]	[Cmol_rhamnolipid_/Cmol_substrate_]	[g_RL_/L]	[h]	[mg_RL_/(Lh)]	[mg_RL_/(Lh)]	[g/(g_CDW _h)]	
*P. aeruginosa *PAO1	MS	Sunflower Oil	250	16.3	0.07	39.00	90	16.67	433.33	0.027	Müller *et al*. 2010b [20]

*P. putida *KCTC 1067	MS	Soybean Oil	20	3.2	0.17	7.30	72	3.90	101.39	0.031	Cha *et al*. 2008 [30]
*P. aeruginosa *PEER02	MS	Soybean Oil	20	not given	0.04	1.82	96	0.73	18.95	not calculable	Wang *et al*. 2007 [28]
*E. coli *HB101	LB	Oleic Acid	4	not given	0.01	0.05	24	0.08	2.18	not calculable	Cabrera-Valladares *et al*. 2006 [29]
*P. putida *KT42C1	LB	Glucose	10	2.0	0.23	1.50	42	1.37	35.71	0.018	This study
*P. aeruginosa *PG201	GS	Glycerol	20	not given	0.17	2.20	168	0.50	13.10	not calculable	Ochsner *et al*. 1995 [27]
*E. coli *TnERAB	LB	Glucose	4	not given	0.07	0.18	24	0.28	7.30	not calculable	Wang *et al*. 2007 [28]
*P. aeruginosa *PEER02	MS	Glucose	20	not given	0.06	0.79	96	0.31	8.18	not calculable	Wang *et al*. 2007 [28]
*P. putida *KT2442	GS	Glycerol	20	1.2	0.05	0.60	25	0.92	24.00	0.020	Ochsner *et al*. 1995 [27]
*E. coli *W3110	M9	Glucose	5	not given	0.04	0.12	48	0.10	2.51	not calculable	Cabrera-Valladares *et al*. 2006 [29]
*E. coli *TnERAB	MS	Glucose	4	not given	0.03	0.08	24	0.12	3.15	not calculable	Wang *et al*. 2007 [28]
*P. fluorescens *ATCC 15453	GS	Glycerol	20	not given	0.02	0.25	168	0.06	1.49	not calculable	Ochsner *et al*. 1995 [27]
*E. coli *DH5α	M9	Glucose	5	not given	< 0.01	< 0.02	168	0.00	0.06	not calculable	Ochsner *et al*. 1995 [27]
*P. oleovorans *GPo1	GS	Glycerol	20	not given	0.00	< 0.02	168	0.00	0.06	not calculable	Ochsner *et al*. 1995 [27]

A comprehensive overview of production performance of recombinant strains is presented. For comparison, wild type P. aeruginosa PAO1 is listed in line 1. The next three lines show data from experiments with hydrophobic substrates in contrast to the remaining entries. This data is based on hydrophilic substrate experiments.

^1^) For the calculation of yields during production on complex media, rhamnolipids were assumed to be synthesized from the used carbon source, while media compounds were utilized for cell growth. ^2^) The specific rhamnolipid production rate was calculated as average over the whole fermentation time. LB, Lysogeny broth medium; MS, mineral salt medium; GS, nitrogen-limited minimal medium.

An important improvement in comparison to the fermentation of *P. aeruginosa *is the utilization of glucose as substrate instead of hydrophobic substances such as plant oils (despite the theoretical yield benefits) (Figure 3). Notably, the carbon yield of 0.23 Cmol_rhamnolipid_/Cmol_glucose _on glucose here reported is 33% of the theoretical yield achievable on this substrate and in the same order as the highest yield on oily carbon substrate reported for *P. aeruginosa *(0.33 Cmol_rhamnolipid_/Cmol_soybean oil_) [53]. The main advantage nevertheless is that purification of rhamnolipids from the fermentation broth can be significantly simplified, as the surfactant emulsifies the hydrophobic substrate in the *P. aeruginosa *fermentation in the aqueous phase. Avoidance of stable substrate/product emulsions using glucose as carbon source results in modest demands on sample preparation for analytical procedures and more importantly in reduced complexity of downstream processing.

Another benefit is that most bacteria grow faster with glucose as carbon source than with fatty acids. Particularly *P. putida *features a high growth rate when growing on glucose [34]. Furthermore, glucose as carbon source is a common substrate in biotechnological production processes, which makes it notably cheaper than for example soybean oil [54]. These advantages of glucose as carbon source for rhamnolipid production outweigh the downside that the theoretical yield is higher with fatty acids as substrate.

### Comparison to recombinant strains

The majority of experiments using recombinant bacteria described so far utilize glucose as substrate for heterologous rhamnolipid production, while some reports with hydrophobic substances exist (Table 1). Disregarding the downsides accompanying the introduction of oily substances in the production process, these strains feature a major advantage. Like the wild type strain *P. aeruginosa *PAO1 they show higher titers than recombinant strains using glucose as carbon source. However, probably due to complex quorum sensing based regulation, these strains need 1 to 3 days to reach the maximal titer. In contrast, and partly also because sugars as carbon source can be taken up and metabolized faster than fatty acids most of the recombinant strains, which constitute a serious alternative to *P. aeruginosa *using hydrophilic substances as substrate reach the maximal concentration of rhamnolipids in the medium after 1 to 2 days. This reduction in process time results in an enhancement of the space-time yield. Notably, except *P. putida *KCTC 1067 [30], the strain engineered in this study features the highest reported space-time yield, making it appropriate for further industrial development of a biotechnological rhamnolipid production.

Comparing strain *P. putida *KT42C1 pVLT31_*rhlAB *further to other attempts of recombinant rhamnolipid production based on hydrophobic substrates, we achieved a two to ten times higher titer (1.5 g/L), which contributes to simplifying downstream processing and entails reduction of investment costs as a smaller reactor is required. The highest carbon yield reported with recombinant strains by now was 0.17 Cmol_rhamnolipid_/Cmol_substrate _[27,30]. The newly engineered strain *P. putida *KT42C1 pVLT31_*rhlAB *had a comparatively high yield of 0.23 Cmol_rhamnolipid_/Cmol_substrate_, which is in the range of yields achieved with *P. aeruginosa *(0.33 Cmol_rhamnolipid_/Cmol_substrate_) [53].

### Logistic growth

Logistic growth, as it can be observed in our experiments (Figure 8) is based on population dynamics. This model is applied in growth situations where the increase in population size is limited by upper boundaries. The general shape of a growth curve is sigmoidal, resulting from zero growth at the beginning, increasing growth to a maximal growth rate and subsequent decreasing growth rates until an asymptote is reached. The logistic model can be utilized to describe such courses. The parameters incorporated in the equation on which logistic growth is based upon resemble the biological parameters of the population. Parameters used are the initial biomass concentration, the final biomass concentration, the time after which half of the biomass is formed, and furthermore a curve form coefficient, which has no direct biological counterpart.

Growth of *P. putida *while producing rhamnolipids can accurately be characterized via a logistic model, as was shown for *P. aeruginosa *[55]. Having in mind the phenomenon of multiauxic growth described earlier, it could be that under the chosen cultivation conditions *P. putida *is experiencing multiple, shifting limitations. One can speculate that these growth-limits are determined by the availability of the currently metabolized carbon source, which changes frequently, when *P. putida *grows on LB-medium supplemented with glucose. Again, the significant reduction in growth is independent of rhamnolipid production.

## Conclusions

In this study, we successfully carried out mono-rhamnolipid synthesis by engineered *P. putida*. Featuring high resistance against rhamnolipids, simple and controllable production kinetics, and the metabolic ability to produce rhamnolipids with high yield and rate, *P. putida *proved to be an appropriate host for heterologous rhamnolipid production.

The fermentation of non-pathogenic *P. putida *was possible using glucose as substrate for the synthesis of mono-rhamnolipids, while constituents of LB-medium were the carbon sources for cell growth. By using quantitative fermentation kinetics monitoring including advanced off-gas analysis we could show that rhamnolipid production is uncoupled from biomass formation, which offers possibilities for substrate exploitation and process management.

In summary, an alternative, non-pathogenic host for rhamnolipid production utilizing glucose as carbon source was successfully developed. The insights into rhamnolipid production with *P. putida *will contribute to the further advance of this host; with the aim to develop an industrially viable process. This study thus adds to an increasing amount of literature regarding industrial importance [56] and the applicability for heterologous expression of complex secondary metabolites [57] of this bacterium.

## Methods

### Bacterial strains, culture conditions, and plasmids

The used bacteria strains *Pseudomonas aeruginosa *PAO1 and *Pseudomonas putida *KT2440 [31,58] were routinely cultivated in LB-medium (10 g/L tryptone, 5 g/L yeast extract, 10 g/L NaCl), while *Escherichia coli *DH5α [59], *Bacillus subtilis *TEB1030 [32], and *Corynebacterium glutamicum *ATCC 13032 [60] were cultivated in TB-medium (12 g/L tryptone, 24 g/L yeast extract, 5 g/L glycerol, 12,54 g/L dipotassium phosphate, 2,31 g/L monopotassium phosphate). All bacteria were cultivated at 37°C except *P. putida *and *C. glutamicum*, which were grown at 30°C. *P. putida *and *E. coli *containing the vector pVLT33 [61] and derivatives thereof were selected by adding 50 μg/mL kanamycin to LB-agar and liquid cultures. For selecting pVLT31 and derivates tetracycline with concentrations of 10 μg/mL for recombinant *E. coli *and 20 μg/mL for recombinant *P. putida *were added. Rhamnolipid production with *P. aeruginosa *and recombinant *P. putida *was carried out using LB-medium complemented with 10 g/L glucose.

### Rhamnolipid toxicity determination

The experiments were carried out with a micro bioreactor system (BioLector, m2p-labs GmbH, Baesweiler, Germany), in 48 well plates (Flowerplates m2p-labs GmbH, Baesweiler, Germany). The biomass concentration was quantified by online light scattering. All bacteria apart from *P. putida *KT2440 were cultivated in 800 to 1000 μL TB-medium. *P. putida *KT2440 was grown in 500 μL LB-Medium supplemented with 10 g/L glucose and 90 mM potassium phosphate buffer (pH 7.4). The growth medium contained rhamnolipid concentrations between 0 g/L and 90 g/L. The cultures were shaken at 1,100 to 1,200 rpm (throw: 3 mm) and incubated at 37°C or 30°C, for *E. coli *DH5α, *B. subtilis *TEB1030, *C. glutamicum *ATCC13032, and *P. putida *KT2440, respectively.

### Construction of the rhamnolipid production module

The *rhlAB *operon was amplified from the genomic DNA of *P. aeruginosa *PAO1 that was isolated with a DNA isolation Kit (DNeasy Blood and Tissue Kit, QIAGEN, Hilden, Germany), using DNA polymerase (*Pfu*Turbo, Stratagene, Waldbronn, Germany) as described by the supplier. The used primer had the following sequences: sense 5'TTGAATTCCATCGGCTACGCGTGAACACGG'3, antisense 5'TTTTTCTAGATCAGGACGCAGCCTTCAGCC'3. The oligonucleotides were obtained from Eurofins MWG Operon (Ebersberg, Germany). The *rhlAB *PCR product was digested with *Eco*RI/*Acc*65I and subsequently ligated into pVLT33, which was digested with the same enzymes, creating the plasmid pVLT33_*rhlAB*. Restriction enzymes and T4 DNA ligase were obtained from Fermentas (St. Leon-Rot, Germany) and used as recommended. DNA manipulation was carried out as described in Sambrook and Russell [62]. Ligations were transformed into competent *E. coli *DH5α using a standard protocol [59]. Transformed cells were selected on LB-agar plates containing 50 μg/mL kanamycin. Experiments with the kanamycin resistant single gene deletion strain *P. putida *KT42C1, lacking the poly(3-hydroxyalkanoic acid) synthase 1 encoded by *phaC1*, required subcloning of *rhlAB *into pVLT31, which contains a gene for tetracycline resistance.

*P. putida *KT2440 was transformed using electroporation as described by Choi *et al*. [63]. Cells containing plasmid pVLT33 or the derivate pVLT33_*rhlAB *were selected on LB-Agar plates or liquid cultures containing 50 μg/mL kanamycin. A resulting strain was denominated *P. putida *KT2440 pVLT33_*rhlAB*. This strain was utilized for all further works.

### Characterization of rhamnolipid production by *P. putida *KT2440 pVLT33_*rhlAB*

For the production of rhamnolipids a main culture of 50 mL LB-medium supplemented with 10 g/L glucose and 50 μg/mL kanamycin in a 500 mL Erlenmeyer flask was inoculated with 1 ml from a starter culture and incubated at 30°C and 200 rpm (throw: 25 mm). The expression of *rhl-*genes was induced by adding IPTG (isopropyl β-D-1-thiogalactopyranoside) to a final concentration of 0.4 mM (guaranteeing full induction) from the beginning of the fermentation. Rhamnolipids were extracted 24 h after induction.

*P. aeruginosa *was cultivated in 10 mL phosphate-limited protease peptone-glucose-ammonium salt medium (PPGAS) at pH 7.2, which promotes the production of rhamnolipids [64], containing 5 g/L glucose, 10 g/L peptone 0.02 M NH_4_Cl, 0.02 M KCl, 0.12 M Tris-HCl, and 0.0016 M MgSO_4_. After 24 h at 37°C, with an agitation of 150 rpm, rhamnolipids were harvested.

Cultivations of *P. putida *KT2440 pVLT33_*rhlAB *carried out in order to supply rhamnolipid-characterization via thin layer chromatography (TLC) and HPLC-ESI-MS featured slightly different process parameters. Only 10 ml of LB-Medium, supplemented with 10 g/L glucose and 50 μg/mL kanamycin in a 100 mL Erlenmeyer flask, were inoculated with an OD_580 _of 0.05 from a starter culture and incubated at 30°C and 150 rpm (throw: 25 mm). IPTG was added to a final concentration of 0.4 mM at an OD_580 _of 0.5.

To gain further insight into the fermentation kinetics of rhamnolipid producing *P. putida *experiments in 300 mL bioreactors (RALF, Bioengineering AG, Wald, Switzerland) were carried out. During 22 hours, the off-gas was analyzed with an off-gas sensor (BlueSens GmbH, Herten, Germany) applying dual wavelength infrared light. This sensor facilitated the simultaneous quantification of ^12^CO_2 _and ^13^CO_2 _concentrations in the off-gas. The reactor was filled with 200 mL LB-medium complemented with 10 g/L 100% labeled ^13^C_6_-glucose, IPTG to a final concentration of 0.4 mM, and 20 μg/mL tetracycline. The temperature was adjusted to 30°C and the aeration rate to 12.1 NL/h. The stirrer speed was set to 500 rpm and after seven hours of fermentation increased to 750 rpm.

The scale-up of rhamnolipid production was tested in a 3.2-liter fermenter vessel (KLF 2000, Bioengineering AG, Wald, Switzerland) with a working volume of 2 liters. The fermenter contained two 6-blade turbine stirrers, a temperature control, a pH control, and a gas inlet. The operating conditions were set to pH 6.8 and a temperature of 30°C, a constant gassing rate of 0.5 vvm and a stirrer speed in the range from 300 to 900 rpm depending on the online-determined pO_2 _signal. Additional glucose was fed using a peristaltic pump.

### Quantification of rhamnolipids

For analysis, rhamnolipids were extracted using 100 μL for orcinol-assay and 500 μL for TLC respectively of cell-free culture broth and 500 μL of ethyl acetate. Samples were mixed by vortexing, with a subsequent phase separation by centrifugation in a tabletop centrifuge at maximum speed (30 sec). The upper, rhamnolipid-containing phase was transferred to a new reaction tube. This procedure was repeated three times. Finally, the organic solvent was removed by evaporation in a vacuum centrifuge.

#### Thin layer chromatography of rhamnolipids

For detection of rhamnolipids using TLC, the dried rhamnolipids were dissolved in 10 μL ethanol. 5 μL of this solution were spotted on a silica 60 TLC-plate (Macherey-Nagel, Düren, Germany). In addition, 5 μL of a 0.1% commercial rhamnolipid extract (JBR425, Jeneil Biosurfactant Co., LCC, Saukville, USA) containing mono- and di-rhamnolipids was spotted. The running buffer was a mixture of chloroform, methanol, and acetic acid in a ratio of 65:15:2. To visualize the rhamnolipids on the TLC-plates, the plates were covered with a detection agent consisting of 0.15 g orcinol, 8.2 mL sulfuric acid (60%), and 42 mL deionized water. For preservation, dried plates were incubated at 110°C for 10 min.

#### Rhamnolipid quantification using orcinol assay

The total amount of rhamnolipids was determined using the orcinol assay [65,66]. The evaporated rhamnolipids were dissolved in 100 μL deionized water. Subsequently 100 μL orcinol solution (1.6% orcinol in deionized water) and 800 μL sulphuric acid (60%) were added. The samples were incubated at 80°C for 30 min and 1000 rpm orbital shaking in a thermomixer (Eppendorf AG, Hamburg, Germany). After cooling to room temperature, the samples were measured at 421 nm in comparison to different concentrations of the commercial rhamnolipid extract using a Genesys 10 UV spectrophotometer (Thermo Fisher Scientific, Waltham, USA).

#### Rhamnolipid quantification using RP-HPLC-CAD

Reversed phase high performance liquid chromatography corona charged aerosol detection (RP-HPLC-CAD) was used for rhamnolipid quantification. Culture samples were centrifuged at 17,700 × g for 30 minutes. 100 μL supernatant were added to 900 μL deionized water, mixed on a vortex shaker, and analyzed on a gradient quaternary reversed phase HPLC system (LaChrom, VWR- Hitachi, Darmstadt, Germany). The system was equipped with an integrated C8(2) silica based column (Luna C8(2), 4.6 × 150 mm, 5 μ, 100 Å, Phenomenex, Inc. Torrance, CA, USA) and a corona CAD (ESA Biosciences Inc., MA, USA). The sample volume was set to 20 μL. The sample was eluted at a flow rate of 800 μL per minute and the temperature of the column oven was set to 40°C. The mobile phase contained filtered water with 0.4% trifluoracetic acid (TFA) (solvent A), acetonitrile (solvent B), and methanol with 0.2% TFA (solvent C) in different ratios. The method started with 20:0:80 (Vol.-% of solvent A:Vol.-% of solvent B:Vol.-% of solvent C) and switched at 7.5 minutes to 2:18:80 during 2 minutes. After 20.6 minutes, the starting concentration was reestablished, again during 2 minutes. The method ended after 24.6 minutes.

#### Rhamnolipid composition characterization by HPLC-ESI-MS

High performance liquid chromatography electrospray ionization mass spectrometry (HPLC-ESI-MS) was used for rhamnolipid characterization (Central Division of Analytical Chemistry/BioSpec, Forschungszentrum Jülich, Jülich, Germany). Rhamnolipids were extracted from 1 L culture broth (5 L Erlenmeyer flask) as described by Déziel *et al*. [8] with small modifications. Cells were removed by centrifugation for 30 min at 9,000 × g and 10°C. The supernatant was acidified with 37% HCL to a pH of 3 and incubated overnight at 4°C. The precipitated rhamnolipids were recovered by centrifugation (9,000 × g, 45 min, 4°C) and resuspended in 15 mL acidified water (pH 3). This suspension was extracted three times with 15 mL ethyl acetate. The combined organic phases were evaporated in a vacuum centrifuge. The residue was dissolved in 15 mL of 0.05 M NaHCO_3_, acidified to pH 2 with 37% HCl, and incubated overnight at 4°C. The precipitate was finally recovered by centrifugation for 60 min at 13,000 × g and 4°C.

For characterization, a binary HPLC system (Agilent 1100 series, Agilent Technologies, Waldbronn, Germany), assembled with a diode array detector (DAD) (190-400 nm), coupled with a triple quadrupole mass spectrometer (4000QTRAP™, Applied Biosystem/MDS SCIEX, Foster City, CA, USA) assembled with a turbo ion spray source was used.

For rhamnolipid separation, normal phase chromatography was used with column dimensions of 150 × 2 mm i.d., 3 μm particle size (ProntoSIL 120-C8-SH, Bischoff Chromatography, Leonberg, Germany) at 20°C. The gradient elution was done with deionized water with 0.1% formic acid (solvent A) followed by different concentrations of acetonitrile with 0.1% formic acid (solvent B). The elution started with 60% B isocratic for 4 min, from 4 to 24 min a linear increase from 60% B to 90% B was applied, subsequently followed by a second isocratic step (90% B for 10 min), and ended by a return to 60% B in one min. The re-equilibration was done with 60% B isocratic for 10 min. All steps were performed at a constant flow rate of 300 μL/min. The injection volume was 20 μL.

The MS was used in negative enhanced mass spectrum mode scanning from 200 - 1000 Da. A flow injection analysis with a standard was used at first to optimize the following parameters: IS -4500 V, declustering potential -100 V, curtain gas (N_2_) 10 arbitrary units (au), source temperature 500°C, nebulizer gas (N_2_) 50 au, and heater gas (N_2_) 20 au. Collision energy (CE) and third quadrupole-entry barrier were set to -5 V and 8 V, respectively. The negative enhanced product ion scan mode was used for structural elucidation MS/MS experiments, in which product ions are generated in the second quadrupole by collision-activated dissociation of selected precursor ions of the first quadrupole and mass analyzed in a linear ion trap. The CE ranged from 30 to 70 V.

The di-rhamnolipid standard (Rha-Rha-C_10_-C_10_) for HPLC analysis was a gift from Sanofi-Aventis Deutschland GmbH, former Hoechst AG (Frankfurt, Germany). Mono-rhamnolipid standard (Rha-C_10_-C_10_) was prepared as described before [67].

#### Rhamnolipid purification by adsorption

The medium was centrifuged in 200 mL cups for 60 min at 4,000 rpm (5810R Eppendorf AG, Hamburg, Germany) to remove cells and cell debris. The cell-free medium was loaded with five times the bed volume per hour by a peristaltic pump (MP-3 Micro Tube Pump, Eyela Inc., Tokyo, Japan) as specified by the manufacturer to a column packed with 90 g of conditioned hydrophobic polymeric adsorbent (Amberlite XAD-2, Sigma-Aldrich, St. Louis, MO, USA). After washing with bidistilled water, rhamnolipids were eluted with 99% isopropanol using a continuous flow (HPLC pump 114 M, Beckman Coulter, Inc., Brea, CA, USA). The organic solvent was evaporated in a freeze dryer (Alpha I-5, Martin Christ Gefriertrocknungsanlagen GmbH, Osterode am Harz, Germany).

### Theoretical capacity estimation

The flux balance analysis was carried out using the software Insilico Discovery (version 3.2.0, Insilico Biotechnology AG, Stuttgart, Germany). The provided metabolic network used for simulations was modified to represent the reaction network of *P. putida *(Additional file 1).

The following reactions were added to the *P. putida *model:

(A) $$\begin{array}{c} \alpha -\text{D}-\text{Glucose}-1-\text{phosphate}+\text{NADPH}+{\text{H}}^{+}+\text{dTTP}\Rightarrow \\ \;\;\;\;\text{dTDP}-\text{L}-\text{rhamnose}+\text{P}{\text{P}}_{i}+\text{NAD}{\text{P}}^{+}+{\text{H}}_{\text{2}}\text{O} \end{array}$$

(B) $$\begin{array}{c} \;\;\;\;2\;\beta -3-\text{hydroxydecanoyl}-\text{ACP + }{\text{H}}_{\text{2}}\text{O}\Leftrightarrow \\ \beta -3-\text{hydroxydecanoyl}-\beta -3-\text{hydroxydecanote + 2}\;\text{ACP} \end{array}$$

(C) $$\begin{array}{c} \text{dTDP}-\text{L}-\text{rhamnose}+\beta -3-\text{hydroxydecanoyl}-\beta -3-\text{hydroxydecanoate}\Leftrightarrow \\ \;\;\text{rhamnosyl}-\beta -3-\text{hydroxydecanoyl}-\beta -3-\text{hydroxydecanoate}+\text{dTDP} \end{array}$$

A linear optimization for rhamnolipid-production with simultaneous minimization of total fluxes was carried out. The rhamnolipid production rate was simulated with different carbon substrates (glucose, glycerol, sucrose, and octanoate). To ensure comparability of the results, the unit Cmol, which normalizes the rhamnolipid production rate to the amount of carbon atoms present in the carbon substrate was chosen. The substrate uptake was varied between 0 and 120 mCmol/(g_CDW _h). The maintenance metabolism, characterized through the simple reaction of ATP to ADP, was varied in the range of 0 to 50 mmol/(g_CDW _h). Blank *et al*. [34] described a value for the non-growth associated maintenance of 10.2 mmol ATP/(g_CDW _h) for *P. putida *DOT-T1E. The considerably higher upper limit of 50 mmol ATP/(g_CDW _h) accounts for scenarios of extra stress, e.g., for metabolic cost of handling high rhamnolipid concentrations. The chosen values for the growth rates were 0 1/h, 0.4 1/h and 0.8 1/h, reflecting ideal production condition, growth observed during rhamnolipid production, and maximal growth of *P. putida *on glucose [68]. Additionally all occurring fluxes were limited to a maximal value of 120 mCmol/(g_CDW _h). Furthermore, variation of the fluxes through the pathways ED pathway, TCA cycle, and PP pathway were examined. In addition, an alternative glucose uptake system, the phosphotransferase system, and a complemented EMP pathway (insertion of a phosphofructokinase reaction for example encoded on a fructose utilization operon by *fruK *(PP0794), catalyzing the conversion of glucose-6P to glucose-1,6P) were simulated.

### Determination of fermentation kinetics

The growth kinetic was described mathematically using a logistic growth model. Logistic growth of pseudomonads had been previously reported for rhamnolipid producing wild type *P. aeruginosa *growing on sunflower oil [55].

The biomass concentration *X *was described using equation 1, where *X_0 _*is the initial biomass concentration, *X_add _*the additional biomass concentration, *t_0 _*the time after which half of *X_add _*is formed, and *b *is a curve form coefficient.

(1) $$X\left(t\right)=X_{0}+\frac{X_{add}}{1+{\left(\frac{t}{t_{0}}\right)}^{b}}$$

The experimental data for the rhamnolipid and glucose concentrations could be described with equations 2 and 3, where *r_RL _*is the specific rhamnolipid production rate [g_rhamnolipid_/(g_CDW _h)] and *r_Glucose _*is the specific glucose uptake rate [g_glucose_/(g_CDW _h)].

(2) $$\frac{dc_{RL}}{dt}=r_{RL}\cdot X$$

(3) dcGlu cosedt=rGlu cose⋅X

A multivariable least squares fit was used to illustrate the development of all three fermentation parameters depending on each other.

Prior to utilizing the described procedure, to fit glucose and rhamnolipid concentrations, two more attempts applying different models were carried out (Additional file 2).

## List of abbreviations used

ACP: Acyl carrier protein; HAA: 3-(3-hydroxyalkanoyloxy)alkanoate; au: Arbitrary units; CDW: Cell dry weight; CE: Collision energy; CoA: Coenzyme A; DAD: Diode array detector; ED: Entner-Doudoroff; EMP: Embden-Meyerhof-Parnas; HPLC-ESI-MS: High performance liquid chromatography electrospray ionization mass spectrometry; IPTG: Isopropyl β-D-1-thiogalactopyranoside; LB: Lysogeny broth; PHA: Polyhydroxyalkanoate; PP: Pentose phosphate; PPGAS: Protease peptone-glucose-ammonium salt medium; RP-HPLC-CAD: Reversed phase high performance liquid chromatography corona charged aerosol detection; TCA: Tricarboxylic acid; TFA: Trifluoracetic acid; TLC: Thin layer chromatography.

## Competing interests

The authors declare that they have no competing interests.

## Authors' contributions

AW carried out the molecular biology and the early strain characterization and drafted parts of the manuscript, TT performed the computational flux balance analysis and drafted most of the manuscript, TTA carried out the growth experiments and fermentation kinetic analysis and drafted parts of the manuscript, PW and JH executed the toxicity experiments and also participated in drafting the manuscript, CM coordinated the toxicity experiments and critically read the manuscript, BK carried out the fermentations and some downstream processing experiments, RW coordinated the fermentations and the downstream processing and critically read the manuscript, MZ executed the preparation of the mono- and di-rhamnolipid standards including fermentation procedures and downstream processing, SW took part in initiating the research done for this manuscript and also in coordination of molecular biologic cloning experiments and critically read the manuscript, RH coordinated the preparation of the standards and took part in drafting the manuscript, CS read the manuscript, FR initiated the research and coordinated the molecular biologic cloning experiments and critically read the manuscript, and LMB coordinated the growth experiments and the flux balance analysis, critically read the manuscript, and designed and coordinated the study. All authors read and approved the final manuscript.

## Supplementary Material

Additional file 1 **Tabular presentation of the in silico reaction network of *Pseudomonas putida***. A list of all reactions implemented in the in silico model of rhamnolipid producing *P. putida*. Click here for file

Additional file 2 **Calculations and assumptions carried out in order to justify the supposition that rhamnolipid production is uncoupled from growth**. Detailed presentation of the calculations carried out and the assumptions made prior to align the equations described in the present work leading to the conclusion that rhamnolipid production is independent of growth. Click here for file
