# Supplementary material for: Growth independent rhamnolipid production from glucose using the non-pathogenic Pseudomonas putida KT2440
Source: Microb Cell Fact. 2011 Oct 17;10:80. doi: 10.1186/1475-2859-10-80 (PMC3258213; doi:10.1186/1475-2859-10-80)
Supplement: Additional file 1 — Tabular presentation of the in silico reaction network of Pseudomonas putida. A list of all reactions implemented in the in silico model of rhamnolipid producing P. putida. [file 1475-2859-10-80-S1.PDF]

Additional file 1: List of in silico reaction network of rhamnolipid producing *Pseudomonas putida*

Identifier	Path-way	Enzyme(s)	Reaction
6PG_hydro-lyase	ED	6-Phospho-D-gluconate hydro-lyase	6-Phospho-D-gluconate => 2-Dehydro-3-deoxy-D-gluconate_6-phosphate + H2O
ABC_Glc			beta-D-Glucose_Fermenter + ATP_Cytosol + H2O_Cytosol => beta-D-Glucose_Cytosol + ADP_Cytosol + Orthophosphate_Cytosol + h_Cytosol
ACK	AC	acetate kinase	Acetyl_phosphate + ADP = Acetate + ATP
ADK	NN	adenylate kinase	AMP + ATP = 2*ADP
AHyd	-		H2O + ATP => ADP + Orthophosphate + H+
AKDH	TCA	alphaketoglutarate dehydrogenase complex	2-Oxoglutarate + NAD + CoA => Succinyl-CoA + NADH + CO2
AMPS	NN		IMP + L-Aspartate + ATP => AMP + ADP + Orthophosphate + Fumarate + H+
ATP:dTDP	-		dTDP + ATP => ADP + dTTP
AicarS	NN		PRPP + 2*L-Glutamine + Glycine + 5*ATP + L-Aspartate + 10-Formyltetrahydrofolate + 4*H2O + CO2 => AICAR + 5*ADP + 7*Orthophosphate + 2*L-Glutamate + Tetrahydrofolate + Fumarate + 9*H+
AkiS	AA		2*Pyruvate + NADPH + 2*H+ => 3-Methyl-2-oxobutanoate + CO2 + NADP + H2O
AlaR	AA	alanine racemase	L-Alanine = D-Alanine
AlaS	AA		Pyruvate + L-Glutamate = L-Alanine + 2-Oxoglutarate
Aldo	EMP	fructose bisphosphate aldolase class I	D-Fructose_1,6-bisphosphate = Glycerone_phosphate + D-Glyceraldehyde_3-phosphate
ArgS	AA		L-Ornithine + Carbamoyl_phosphate + L-Aspartate + ATP + H2O = L-Arginine + Fumarate + AMP + 3*Orthophosphate + 4*H+
AsnS	AA		L-Aspartate + NH4 + ATP + H2O => L-Asparagine + 2*Orthophosphate + AMP + H+
AspS	AA		Oxaloacetate + L-Glutamate = L-Aspartate + 2-Oxoglutarate
BioS			25.82*Protein + 10.94*DNA + 38.76*RNA + 1.46*Lipid + 19.36*polys + 3.66*Mur = bio
CDPK	NN		CTP + ADP = CDP + ATP
CMPK	NN		CDP+ ADP = CMP + ATP
CSA	TCA	citrate synthase and aconitase	Oxaloacetate + Acetyl-CoA + H2O = Isocitrate + CoA + H+
CTPS	NN		UTP + L-Glutamine + ATP + H2O => CTP + ADP + Orthophosphate + 2*H+ + L-Glutamate
CarpS	AA		2*H2O + CO2 +L-Glutamine + 2*ATP => Carbamoyl_phosphate + L-Glutamate + Orthophosphate + 2*ADP + 3*H+
ChoS	CB		2-Dehydro-3-deoxy-D-arabino-heptonate_7-phosphate + Phosphoenolpyruvate + NADPH + ATP => Chorismate + ADP + 3*Orthophosphate + NADP + H2O
CysS	AA		L-Serine + Acetyl-CoA + H2S => L-Cysteine + CoA + Acetate + H+
DAHPS	CB	2-dehydro-3-deoxyphosphoheptonate aldolase	Phosphoenolpyruvate + D-Erythrose_4-phosphate + H2O => 2-Dehydro-3-deoxy-D-arabino-heptonate_7-phosphate + Orthophosphate
DG6P	ED	2-dehydro-3-deoxy-D-gluconate-6-phosphate	2-Dehydro-3-deoxy-D-gluconate_6-phosphate => D-Glyceraldehyde_3-phosphate + Pyruvate
DipS	AA	diaminopimelate biosynthesis	L-Aspartate + Pyruvate + L-Glutamate + Succinyl-CoA + ATP + 2*NADPH + H+ => LL-2,6-Diaminoheptanedioate + 2-Oxoglutarate + Succinate + CoA + ADP + 2*NADP + Orthophosphate
ENO	EMP	enolase	D-Glycerate_2-phosphate = Phosphoenolpyruvate + H2O
FADHO	OP	fadh2 oxidation	FADH2 + CoQ => CoQH2 + FAD

FATP	OP	F1F0 ATPase	H+_out + 0.25*ADP + 0.25*Orthophosphate + 0.25*H+ => H_in + 0.25*ATP + 0.25*H2O
FBP	EMP	fructose-bisphosphatase	D-Fructose_1,6-bisphosphate + H2O => beta-D-Fructose_6-phosphate + Orthophosphate
FDH	-	formate dehydrogenase	Formate + NAD = NADH+ CO2
FU	TCA	fumarase	Fumarate + H2O = L-Malate
G1PAT	PB	ADP:alpha-D-glucose-1-phosphate adenylyltransferase	alpha-D-Glucose_1-phosphate + ATP => ADPglucose + Pyrophosphate
G3PDH	FA		Glycerone_phosphate+ NADH + H+ = sn-Glycerol_3-phosphate + NAD
G6PDH	PPP	glucose 6-phosphate-1-dehydrogenase und 6-phosphogluconolactonase	beta-D-Glucose_6-phosphate + NADP + H2O => 6-Phospho-D-gluconate + NADPH + 2*H+
GAPDH	EMP	glyceraldehyde 3-phosphate dehydrogenase-A complex	D-Glyceraldehyde_3-phosphate + Orthophosphate + NAD = 3-Phospho-D-glyceroyl_phosphate + NADH + H+
GLK	GA	glucokinase	beta-D-Glucose_6-phosphate + ADP + H+ = beta-D-Glucose + ATP
GMPS	NN		IMP + NAD + ATP + L-Glutamine + 3*H2O => GMP + AMP + NADH + 2*Orthophosphate + 4*H+ + L-Glutamate
GluR	MB	glutamate racemase	L-Glutamate = D-Glutamate
GlumS	AA		L-Glutamate + NH4 + ATP = L-Glutamine + ADP + Orthophosphate
GlutS	AA		NH4 + 2-Oxoglutarate + NADPH + H+ => L-Glutamate + NADP + H2O
Gly	GM		Glycerol + ATP + NAD = D-Glyceraldehyde_3-phosphate + ADP + NADH + 2*H+
GlyS	AA		L-Serine + Tetrahydrofolate = Glycine + 5,10-Methylenetetrahydrofolate + H2O
Glyx	TCA	glyoxylate-shunt	Isocitrate + Acetyl-CoA + H2O => Succinate + L-Malate + CoA + H+
HisS	AA		PRPP + L-Glutamine + ATP + 2*NAD + 5*H2O => L-Histidine + AICAR + 2-Oxoglutarate + 2*NADH + 7*H+ + 5*Orthophosphate
HomS	AA		L-Aspartate + ATP + 2*NADPH + 2*H+ = L-Homoserine + ADP + Orthophosphate + 2*NADP
ICD	TCA	isocitrate dehydrogenase	Isocitrate + NAD = NADH + 2-Oxoglutarate + CO2
IMPS	NN		AICAR + 10-Formyltetrahydrofolate => Tetrahydrofolate + IMP + H2O + 2*H+
Ileus	AA		L-Threonine + Pyruvate + NADPH + L-Glutamate + 2*H+ => L-Isoleucine + NH4 + NADP + H2O + CO2 + 2-Oxoglutarate
LeuS	AA		3-Methyl-2-oxobutanoate + L-Glutamate + Acetyl-CoA + H2O + NAD => L-Leucine + 2-Oxoglutarate + CoA + NADH + H+ + CO2
LysS	AA		LL-2,6-Diaminoheptanedioate + H+ => L-Lysine + CO2
MDH	TCA	malate dehydrogenase	L-Malate + NAD = Oxaloacetate + NADH + H+
MalCx	GN	malate oxidoreductase	L-Malate + NADP => Pyruvate + CO2 + NADPH
MetS	AA		L-Homoserine + Succinyl-CoA + L-Cysteine + 5-Methyltetrahydrofolate + H2O => L-Methionine + CoA + Succinate + Pyruvate + NH4 + H+ + Tetrahydrofolate
MurS	MB		2*beta-D-Fructose_6-phosphate + 2*L-Glutamine + 2*Acetyl-CoA + 7*ATP + Phosphoenolpyruvate + NADPH + L-Alanine + D-Glutamate + LL-2,6-Diaminoheptanedioate + D-Alanine + 5*H2O => Murein_subunit + 2*CoA + 2*L-Glutamate + 6*ADP + AMP + NADP + 11*Orthophosphate + 11*H+
NADHO	OP	NADH oxidation	NADH + H+ + CoQ +4*H_in => CoQH2 + NAD + 4*H_out
Oct	OM		Octanoyl-CoA + 3*CoA + 3*FAD + 3*H2O + 3*NAD => 3*FADH2 + 3*NADH + 3*h + 4*Acetyl-CoA
OctACP	OM		Octanoyl-CoA + ACP => Octanoyl-ACP + CoA
OrnS	AA		2*L-Glutamate + Acetyl-CoA + ATP + NADPH + H2O => L-Ornithine + 2-Oxoglutarate + CoA + ADP + Orthophosphate + NADP+ Acetate
PDH	PD	PD multienzyme complex	Pyruvate + CoA + NAD => Acetyl-CoA + CO2 + NADH
PEPCx	GN	phosphoenolpyruvate carboxylase	Phosphoenolpyruvate + CO2 + H2O = Oxaloacetate + Orthophosphate + H+
PGDH	PPP	6-phosphogluconate dehydrogenase (decarboxylating)	6-Phospho-D-gluconate + NADP => D-Ribulose_5-phosphate + CO2 + NADPH

PGI	EMP	phosphoglucose isomerase	beta-D-Glucose_6-phosphate=beta-D-Fructose_6-phosphate
PGK	EMP	phosphoglycerate kinase	3-Phospho-D-glyceroyl_phosphate + ADP = D-Glycerate_3-phosphate + ATP
PGM	GA	phosphoglucomutase	beta-D-Glucose_6-phosphate = alpha-D-Glucose_1-phosphate
PGluMu	EMP	phosphoglycerate mutase 1	D-Glycerate_3-phosphate = D-Glycerate_2-phosphate
PK	EMP	pyruvate kinase I	Phosphoenolpyruvate + ADP + H+ => Pyruvate + ATP
PPase	OP	anorganische Pyrophophatase	Pyrophosphate + H2O => 2*Orthophosphate
PTA	AC	phosphate acetyltransferase	Orthophosphate + Acetyl-CoA = Acetyl_phosphate + CoA
PYRCx	GN	pyruvate carboxylase	Pyruvate + ATP + HCO3 = Oxaloacetate + Orthophosphate + H+ + ADP
PheS	AA		Chorismate + L-Glutamate + H+ => L-Phenylalanine + 2-Oxoglutarate + CO2 + H2O
ProS	AA		L-Glutamate + ATP + 2*NADPH + H+ => L-Proline + ADP + Orthophosphate + H2O + 2*NADP
R5PI	PPP	ribose-5-phosphate isomerase	D-Ribose_5-phosphate= D-Ribulose_5-phosphate
RPE	PPP	ribulose phosphate 3-epimerase	D-Ribulose_5-phosphate = D-Xylulose_5-phosphate
RPPK	PRB	ribose-phosphate pyrophosphokinase	ATP + D-Ribose_5-phosphate => AMP + H+ + PRPP
SDH	TCA	succinate dehydrogenase	Succinate + FAD = Fumarate + FADH2
SUS	TCA	succinyl-CoA synthetase	Succinyl-CoA + ADP + Orthophosphate = Succinate + CoA + ATP
SerS	AA		D-Glycerate_3-phosphate + L-Glutamate + NAD + H2O = L-Serine + 2-Oxoglutarate + Orthophosphate + NADH + H+
Suc	SM		Sucrose + Orthophosphate + ATP = alpha-D-Glucose_1-phosphate + ADP + beta-D-Fructose_6-phosphate + H+
SulRed	SUM		Sulfate + 2*ATP + 4*NADPH + 4*H+ => H2S + 4*NADP + 2*ADP + 2*Orthophosphate + 2*H2O
TA	PPP	transaldolase	Sedoheptulose_7-phosphate + D-Glyceraldehyde_3-phosphate = beta-D-Fructose_6-phosphate + D-Erythrose_4-phosphate
THG	-	transhydrogenase	NADPH + NAD = NADP + NADH
TIS	EMP	triose phosphate isomerase	Glycerone_phosphate = D-Glyceraldehyde_3-phosphate
TKa	PPP	transketolase, reaction A	D-Ribose_5-phosphate + D-Xylulose_5-phosphate = Sedoheptulose_7-phosphate + D-Glyceraldehyde_3-phosphate
TKb	PPP	transketolase, reaction B	beta-D-Fructose_6-phosphate + D-Glyceraldehyde_3-phosphate = D-Erythrose_4-phosphate + D-Xylulose_5-phosphate
ThrS	AA		L-Homoserine + ATP + H2O = L-Threonine + ADP + Orthophosphate + H+
TrpS	AA		Chorismate + L-Glutamine + PRPP + L-Serine => L-Tryptophan + 2*Orthophosphate + CO2 + D-Glyceraldehyde_3-phosphate + L-Glutamate + Pyruvate + H2O + 2*H+
TyrS	AA		Chorismate + L-Glutamate + NAD => L-Tyrosine + 2-Oxoglutarate + CO2 + NADH
UDPK	NN		UDP + ATP = UTP + ADP
UMPK	NN		UMP + ATP = UDP + ADP
UMPS	NN		Carbamoyl_phosphate + L-Aspartate + 0.5*O2 + PRPP => UMP + H2O + CO2 + 3*Orthophosphate + H+
UO	OP	ubiquinone oxidation	CoQH2 + 0.5*O2 + 4*H+_in => CoQ + H2O + 4*H+_out
ValS	AA		3-Methyl-2-oxobutanoate + L-Glutamate = L-Valine + 2-Oxoglutarate
accADBC_fabD	FA		ACP + Acetyl-CoA + HCO3 + ATP = Malonyl-ACP + CoA + H+ + ADP + Orthophosphate
cynT	-	carbonate dehydratase	H2O + CO2 = HCO3 + H+
dAS	NN		ATP + NADPH + H+ = dATP + H2O + NADP
dCS	NN		CDP + ATP + NADPH + H+ = dCTP + ADP + NADP + H2O
dGS	NN		GMP + 2*ATP + NADPH + H+ = dGTP + 2*ADP + NADP + H2O
dTS	NN		UDP + 3*ATP + 2*NADPH + H2O + 5,10-Methylenetetrahydrofolate => dTTP + 3*ADP + 2*NADP + 2*Orthophosphate + Tetrahydrofolate
fThfS	FM		Tetrahydrofolate + ATP + NADH + CO2 => 10-Formyltetrahydrofolate + ADP + Orthophosphate + NAD
fabABFGIZ	FA		Acetoacetyl-ACP + 7*NADPH + 10*H+ + 3*Malonyl-ACP = 2-trans-Decenoyl-ACP + 7*NADP + 4*H2O + 3*CO2 + 3*ACP
fabABGIZ_161	FA		2-trans-Decenoyl-ACP + 3*Malonyl-ACP + 9*H+ + 6*NADPH = 3*CO2 + 3*ACP + 6*NADP + 3*H2O + cis-Hexadecenoyl-ACP

fabABGI_140	FA		Dodecanoyl-ACP + 3*H+ + Malonyl-ACP + 2*NADPH = CO2 + ACP + 2*NADP + Tetradecanoyl-ACP + H2O
fabAFGI_120	FA		3*H+ + Decanoyl-ACP + Malonyl-ACP + NADH + NADPH = ACP + CO2 + NAD + Dodecanoyl-ACP + NADP + H2O
fabAFGI_160	FA		3*H+ + Tetradecanoyl-ACP + Malonyl-ACP + 2*NADPH = ACP + CO2 + H2O + Hexadecanoyl-ACP + 2*NADP
fabAGHI_50	FA		3*H+ + 2*NADPH + Propionyl-CoA + Malonyl-ACP = CO2 + CoA + 2*NADP + H2O + Pentanoyl-ACP
fabBG	FA		Octanoyl-ACP + NADPH + 2*H+ + Malonyl-ACP = R-3-hydroxydecanoyl-ACP + NADP + CO2 + ACP
fabBGIZ_150	FA		15*h + 10*NADPH + Pentanoyl-ACP + 5*Malonyl-ACP => 5*CO2 + 5*ACP + 10*NADP + 5*H2O + Pentadecanoyl-ACP
fabBGIZ_170	FA		3*H+ + Pentadecanoyl-ACP + Malonyl-ACP + 2*NADPH = ACP + CO2 + H2O + Heptadecanoyl-ACP + 2*NADP
fabBGIZ_181	FA		3*H+ + cis-Hexadecenoyl-ACP + Malonyl-ACP + 2*NADPH = ACP + CO2 + 2*NADP + H2O + cis-Octadecenoyl-ACP
fabGAIB	FA		Acetoacetyl-ACP + 3*NADPH +3*NADH + 8*H+ + 2*Malonyl-ACP => Octanoyl-ACP + 3*NADP + 3*H2O + 2*CO2 + 2*ACP + 3*NAD
fabI_100	FA	2-trans-Decenoyl-[acyl-carrier protein] reductase (NADH)	2-trans-Decenoyl-ACP + NADH + H+ => Decanoyl-ACP + NAD
fab_BH	FA		H+ + Acetyl-CoA + Malonyl-ACP = CO2 + CoA + Acetoacetyl-ACP
fatty	FA	Virtual reaction to average fatty acids	0.04*Dodecanoyl-ACP + 0.11*Tetradecanoyl-ACP + 0.11*Pentadecanoyl-ACP + 0.35*Hexadecanoyl-ACP + 0.15*cis-Hexadecenoyl-ACP + 0.12*Heptadecanoyl-ACP + 0.12*cis-Octadecenoyl-ACP = fatty-ACP
meThfS	FM		NADH + NH4 + CO2 + 5,10-Methylenetetrahydrofolate = NAD + Glycine + Tetrahydrofolate
myThfS	FM		Tetrahydrofolate + CO2 + 3*NADH + 3*H+ => 5-Methyltetrahydrofolate + 3*NAD +2*H2O
pgsA_pgpA	FA		H2O + sn-Glycerol_3-phosphate + CDP-diacylglycerol = CMP + H+ + Orthophosphate + Phosphatidylglycerol
plsB_cdsA	FA		0.5*CTP + 0.5*sn-Glycerol_3-phosphate + fatty-ACP = ACP + 0.5*Pyrophosphate + 0.5*CDP-diacylglycerol
pssA_psd	FA		L-Serine + CDP-diacylglycerol = CMP + CO2 + Phosphatidylethanolamine
sdaA_1	AA	L-threonine deaminase I	L-Threonine => 2-Oxobutyrate + NH4
tdcE_2	AA	2-ketobutyrate formate-lyase	2-Oxobutyrate + CoA = Formate + Propionyl-CoA

constructed Reactions, designed to yield a pool of compounds

DNAPol	2.706*dATP + 2.794*dGTP + 2.706*dTTP + 2.794*dCTP + 10*ATP + 10*H2O = DNA + 10*ADP + 10*Orthophosphate + 10*H+
LipidPol	31.99*Phosphatidylglycerol + 97*Phosphatidylethanolamine = Lipid
MurPol	11*Murein_subunit = Mur + 20*H2O
Protpol	36.41*L-Alanine + 15.97*L-Arginine + 15.97*L-Aspartate + 15.97*L-Asparagine + 5.43*L-Cysteine + 17.88*L-Glutamate + 17.88*L-Glutamine + 27.47*Glycine + 5.43*L-Histidine + 14.69*L-Isoleucine + 29.06*L-Leucine + 17.88*L-Lysine + 7.66*L-Methionine + 10.86*L-Phenylalanine + 13.41*L-Proline + 15.65*L-Serine + 16.93*L-Threonine + 3.51*L-Tryptophan + 8.94*L-Tyrosine + 22.99*L-Valine + 1276*ATP + 1276*H2O = Protein + 1276*ADP + 1276*Orthophosphate + 1276*H+
RNAPol	2.88*AMP + 3.54*GMP + 2.38*UMP + 2.2*CMP + 30*ATP + 30*H2O = RNA + 30*ADP + 30*Orthophosphate + 30*H+
SPol	10*ADPglucose+1*alpha-D-Glucose_1-phosphate=1*polys+10*ADP+10*h

Pathways: AC, Acetate metabolism; AA, Amino acids biosynthesis; CB, Chorismate biosynthesis; TCA, Citrate cycle; EMP, Embden-Meyerhof-Parnas Pathway; ED, Entner-Doudoroff pathway; FA, Fatty acids biosynthesis; FM, Folate metabolism; GN, Gluconeogenesis; GA, Glucose assimilation; GM, Glycerol metabolism; MB, Murein biosynthesis; NM, Nucleotide metabolism; OM, Octanoate metabolism; OP, Oxidative phosphorylation; PPP, Pentose phosphate pathway; PB, Polysaccharide biosynthesis; PRPP, PRPP biosynthesis; PD, Pyruvate dehydrogenase; SM, Sucrose metabolism; SUM, Sulfur metabolism
