# Supplementary material for: Growth independent rhamnolipid production from glucose using the non-pathogenic Pseudomonas putida KT2440
Source: Microb Cell Fact. 2011 Oct 17;10:80. doi: 10.1186/1475-2859-10-80 (PMC3258213; doi:10.1186/1475-2859-10-80)
Supplement: Additional file 2 — Calculations and assumptions carried out in order to justify the supposition that rhamnolipid production is uncoupled from growth. Detailed presentation of the calculations carried out and the assumptions made prior to align the equations described in the present work leading to the conclusion that rhamnolipid production is independent of growth. [file 1475-2859-10-80-S2.PDF]

Additional file 2: Rhamnolipid production is uncoupled from growth

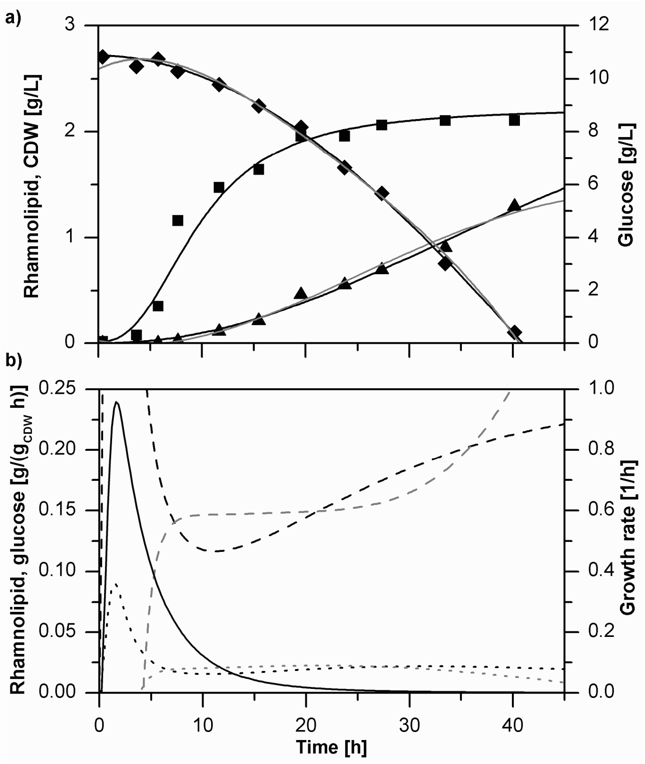

Uncoupling of rhamnolipid production and cell growth of *P. putida*. The same experimental data as in figure 8 is shown but with different mathematical fittings are shown. The curves for CDW and μ are identical to the graphs in figure 8. (a) Fermentation characteristics including cell growth (■) and course of rhamnolipid (▲) and glucose (♦) concentrations and their respective fitted courses. The black lines present a logistic fit, while the grey lines display a polynomial approach. (b) Specific rates resulting from the fitted data. The line represents the course of the growth rate, while the dashed line and the dotted line show the specific glucose uptake rate and the specific rhamnolipid production rate, respectively. Again the black lines present the rates calculated from the logistic fits, while the rates represented with grey lines emanate from calculations with the polynomial fit data. CDW, Cell Dry Weight.

$$Y(t) = A + \frac{B}{1 + \left(\frac{t}{C}\right)^D} \quad (4)$$

$$Y(t) = a \cdot t^4 + b \cdot t^3 + c \cdot t^2 + d \cdot t + e \quad (5)$$

To strengthen our quote that rhamnolipid-production is growth independent and that the specific rhamnolipid production rate is constant throughout the whole experiment, different mathematical models were applied for fitting the experimental data. In a first attempt, a logistic model (Equation 4) was utilized (Figure appendix a, black lines) to fit all three data sets. In a second attempt, a polynomial model (Equation 5) was applied for the course of glucose and rhamnolipid concentrations (Figure appendix a, grey lines). The biomass data was always fitted using the logistic growth model (Equation 1). These fits then were used to calculate the specific glucose uptake rate and the specific rhamnolipid production rate depending on cell dry weight [g/(g_{CDW} h)] (Equations 2 and 3, Figure appendix b). As stated in the results section, the specific rhamnolipid production rate is almost constant, and is not dependent on the fitting procedure used. The same applies to the specific glucose uptake rate. The deviations from an absolute linear course in the beginning (Figure a) result from the low cellular dry weight and the

thus inaccurate fitting in this region. The deviations in the specific glucose uptake rate can be attributed to the chosen polynomic function, which does not fit the data as exact as the logistic function.

Comparing the curves for glucose consumption and rhamnolipid production fitted in the appendix to the calculated curves in the manuscript (Figure 8, Figure appendix a), visualizes an improved fit of the experimental data using the two alternative fitting methods presented above (Equations 4 and 5). The average deviation of the three graphs from the experimental data was calculated by the sum of the squares of the deviation, and summed up to 0.71 in the case of polynomic fits, to 0.40 in the case of logistic fits, and to 2.56 when the time courses were calculated. The small differences using three partially independent fitting procedures justify the application of the procedure described in the materials and methods section to approximate the kinetic time courses from the experimental data.
